# Platelet Reactivity in Individuals Over 65 Years Old Is Not Modulated by Age

**DOI:** 10.1161/CIRCRESAHA.119.316324

**Published:** 2020-04-27

**Authors:** Melissa V. Chan, Ming-Huei Chen, Temo Barwari, Jennifer E. Huffman, Paul C. Armstrong, Shih-Jen Hwang, Peter Santer, Brigitte Wierer, Manuel Mayr, Stefan Kiechl, Andrew D. Johnson, Johann Willeit, Timothy D. Warner

**Affiliations:** 1 From the The Blizard Institute, Barts and The London School of Medicine ’ Dentistry, Queen Mary University of London (M.V.C., P.C.A., T.D.W.); 2 The National Heart, Lung, and Blood Institute’s Framingham Heart Study (M.V.C., M.-H.C., J.E.H., S.-J.H., A.D.J.); 3 King’s British Heart Foundation Centre, King’s College London (T.B., M.M.); 4 Department of Laboratory Medicine, Bruneck Hospital (P.S., B.W.); 5 Department of Neurology, Medical University Innsbruck, Austria (S.K., J.W.).

**Keywords:** aging, aspirin, atherosclerosis, cardiovascular diseases, myocardial infarction, platelet aggregation, risk

**Meet the First Author, see p 332**

Platelet reactivity is a key determinant of arterial thrombosis, as established by a vast amount of research over many years. Despite arterial thrombotic mortality and morbidity risks increasing with age, we noted a remarkable lack of data regarding platelet function in older people. Indeed, while the largest study to date showed no change in platelet reactivity in older individuals, it included only 31 healthy people over the age of 65.^1^ To address this critical gap in understanding, we have assessed platelet reactivity in an elderly population.

The Bruneck Study is a prospective, community-based survey on the epidemiology and pathogenesis of atherosclerosis, and the cohort provides a unique opportunity to study platelet function in the elderly and to discriminate the influences of healthy aging from the effects of cardiovascular disease. The rates of myocardial infarction, stroke, and type II diabetes mellitus within this cohort were significantly lower than the equivalent rates in Italy. Body mass index (25.4±4.1 kg/m^2^) was also lower than the European average published by WHO (26.9 kg/m^2^).

The 338 surviving participants of the Bruneck Study (51% male, aged 65.8–98.9 years) were recalled in April 2016.^2^ The study complied with the Declaration of Helsinki and was approved by the local ethics committees of Bolzano and Verona. All study subjects provided written informed consent. Fasting blood samples were collected for biomarkers or platelet testing. Platelet reactivity was assessed using light transmission aggregometry (LTA) with arachidonic acid (AA), ADP, Horm collagen, TRAP-6 (thrombin receptor activator peptide 6) amide and U46619 as agonists. Data are reported as % final aggregation after 6 minutes. Data were analyzed using Prism 8 (GraphPad) and R. Nonparametric Spearman correlation with Fisher transformation and *t*-tests were performed and correlation coefficient (*r*) was reported.

Platelet number was significantly greater in females than in males (250±66×10^3^/µL versus 204±52×10^3^/µL; *P*<0.0001). We observed no decline in platelet count associated with age in the population in either males or females (male *r*=−0.125, *P*=0.104; female *r*=−0.073, *P*=0.355).

Platelet aggregation following stimulation by an agonist was expressed as median (IQR)% with 0% representing no aggregation and 100% as complete aggregation. Final aggregation at 6 minutes in the Bruneck Study cohort as determined by LTA (median [IQR]%) in response to AA (1 mmol/L, n=337) was 4 (65)%, ADP (5 μmol/L, n=335) 57 (19)%, ADP (20 μmol/L, n=337) 61 (13)%, collagen (0.4 μg/mL, n=332) 38 (55)%, collagen (4 μg/mL, n=333) 61 (12)%, collagen (10 μg/mL, n=337) 61 (12)%, TRAP-6 amide (25 μmol/L, n=337) 63 (9)% and U46619 (10 μmol/L, n=337) 64 (7)%.

Aspirin use was defined by LTA as final aggregation <20% in response to AA (which we propose as a sensitive determinant of aspirin use that is superior to self-report).^3^ Consistent with its effects on cyclooxygenase-1, aspirin abolishes aggregation responses in LTA to AA and reduces aggregation to low concentrations of collagen. When excluding those exposed to aspirin (n=150), final aggregation responses to ADP, collagen, TRAP-6 amide, and U46619 indicated no association with age. AA responses, however, reduced with age (*r*=0.193, n=139, *P*=0.023), which we speculate is because of age-related increases in the use of nonsteroidal anti-inflammatory drugs (Figure). These results reflect the previous smaller study using whole blood aggregometry.^1^

**Figure.**
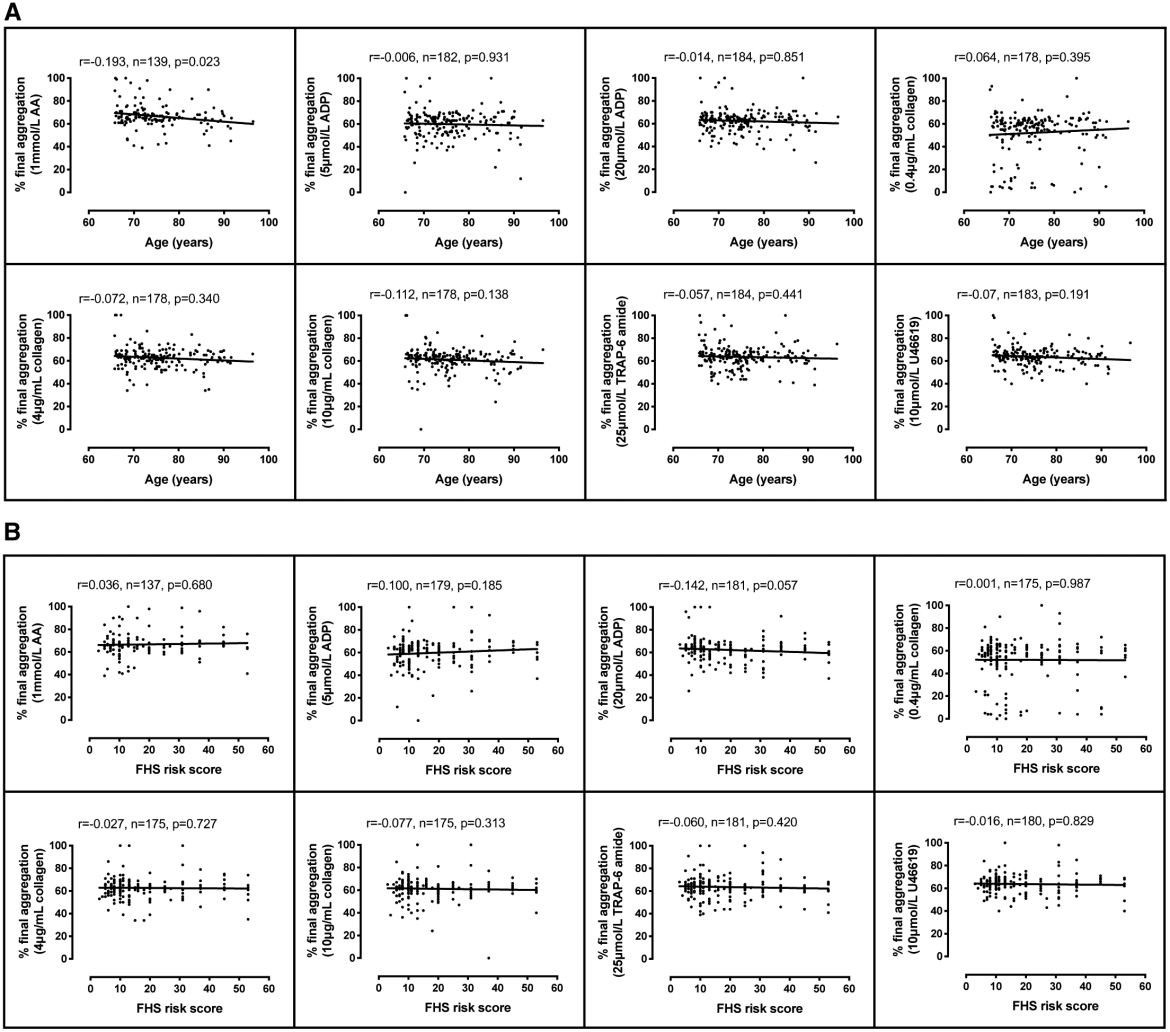
**Platelet responses by aggregometry in Bruneck Study participants.** **A**, Light transmission aggregometry (LTA) determination of % final aggregation at 6 min in response to arachidonic acid (AA; 1 mmol/L), ADP (5 μmol/L), ADP (20 μmol/L), collagen (0.4 μg/mL), collagen (4 μg/mL), collagen (10 μg/mL), TRAP-6 amide (25 μmol/L), and U46619 (10 μmol/L) compared with age (n=139–184) or (**B**) 10-y cardiovascular risk by FHS (Framingham Heart Study) risk score (n=137–181) measured in the absence of aspirin use. *r* indicates the correlation between final aggregation and age or FHS risk score using Spearman correlation.

As platelet reactivity was not altered with older age and the efficacy of aspirin was unchanged, we used FHS (Framingham Heart Study) 10-year predictive risk scores as a tool to assess cardiovascular disease risk status. FHS risk was determined using SAS software (Cary, NC) based on the patient age, sex, systolic blood pressure, use of antihypertensive drugs, diabetes mellitus, smoking status, and total and HDL (high-density lipoprotein) cholesterol levels. Contrary to the expectation that platelet reactivity would be higher in people with greater risk and recent studies relating ADP-induced platelet reactivity and future cardiovascular events,^4^ we demonstrated no association of age with ex vivo platelet reactivity to any agonist in a healthy, older population (Figure).

We recognize some limitations with our study. As with all such investigations into older people, participants are, by definition, healthier survivors. In addition, we were constrained by the blood volume available in the number of agonist concentrations used in LTA, although this study reports the largest number of agonists and concentrations in an elderly population to date. Sensitivity to aspirin was not verified by addition in vitro, similarly due to limited availability of blood. Finally, we acknowledge that this study was under-powered and required 310 nonaspirin takers to make a definitive conclusion.

While mortality associated with cardiovascular disease increases with age and platelets play a clear role in arterial thrombotic events, we present a systematic, comprehensive study in an elderly population demonstrating that ex vivo platelet reactivity does not significantly change with ageing in healthy individuals. We propose that increased risk of arterial thrombotic events with age must therefore be a result of how platelets react to the prothrombotic environment caused by underlying vascular disease (plaque erosion and fissuring) and increased inflammation with age rather than changes in the platelet per se.

Data and supporting materials can be made available upon request from the corresponding authors.

## Acknowledgments

We thank the staff at Bruneck Hospital, in particular the nurses and doctors. We also extend our thanks to the Governor of the Province of Bozen and participants of the Bruneck Study. FHS risk score was calculated using a script provided by Martin Larson, Framingham. The views expressed in this manuscript are those of the authors and do not necessarily represent the views of the National Heart, Lung, and Blood Institute; the National Institutes of Health; or the US Department of Health and Human Services.

## Sources of Funding

This work was supported by grants from the British Heart Foundation (BHF; PG/15/47/31591, an Interdisciplinary PhD Studentship, PG/15/79/31777, CH/16/3/32406 and RG/16/14/32397); the National Heart, Lung and Blood Institute (NHLBI; Division of Intramural Research funds and Contract No. N01-HC-25195 to the FHS in collaboration with Boston University); the National Institute of Health Research Biomedical Research Centre based at Guy’s and St Thomas’ National Health Service (NHS) Foundation Trust; King’s College London in partnership with King’s College Hospital; an excellence initiative VASCage (Centre for Promoting Vascular Health in the Ageing Community); an R&D K-Project (843536); and K-Centre (COMET program—Competence Centers for Excellent Technologies) funded by the Austrian Ministry for Transport, Innovation and Technology, the Austrian Ministry for Digital and Economic Affairs and the federal states Tyrol, Salzburg, and Vienna.

## Disclosures

None.
